# Getting the feel of food structure with atomic force microscopy

**DOI:** 10.1016/j.foodhyd.2017.05.017

**Published:** 2018-05

**Authors:** A. Patrick Gunning, Victor J. Morris

**Affiliations:** Quadram Institute Bioscience, Norwich Research Park, Colney, Norwich, NR4 7UA, UK^1^

**Keywords:** Atomic force microscopy, Colloids, Emulsions, Polysaccharides, Mucin, Ligand-receptor interactions

## Abstract

This article describes the progress in the development of the atomic force microscope as an imaging tool and a force transducer, with particular reference to applications in food science. Use as an imaging tool has matured and emphasis is placed on the novel insights gained from the use of the technique to study food macromolecules and food colloids, and the subsequent applications of this new knowledge in food science. Use as a force transducer is still emerging and greater emphasis is given on the methodology and analysis. Where available, applications of force measurements between molecules or between larger colloidal particles are discussed, where they have led to new insights or solved problems related to food science. The future prospects of the technique in imaging or through force measurements are discussed.

## Highlights

• Ultrastructure of polysaccharides.
• Structure-function relations of food molecules.
• Physical effects of interfacial stabilisers and deformable colloids.
• Bio and physical activity of pectin components.
• Characterisation of glycan distribution on mucin.

## Graphical abstract

**Figure:**
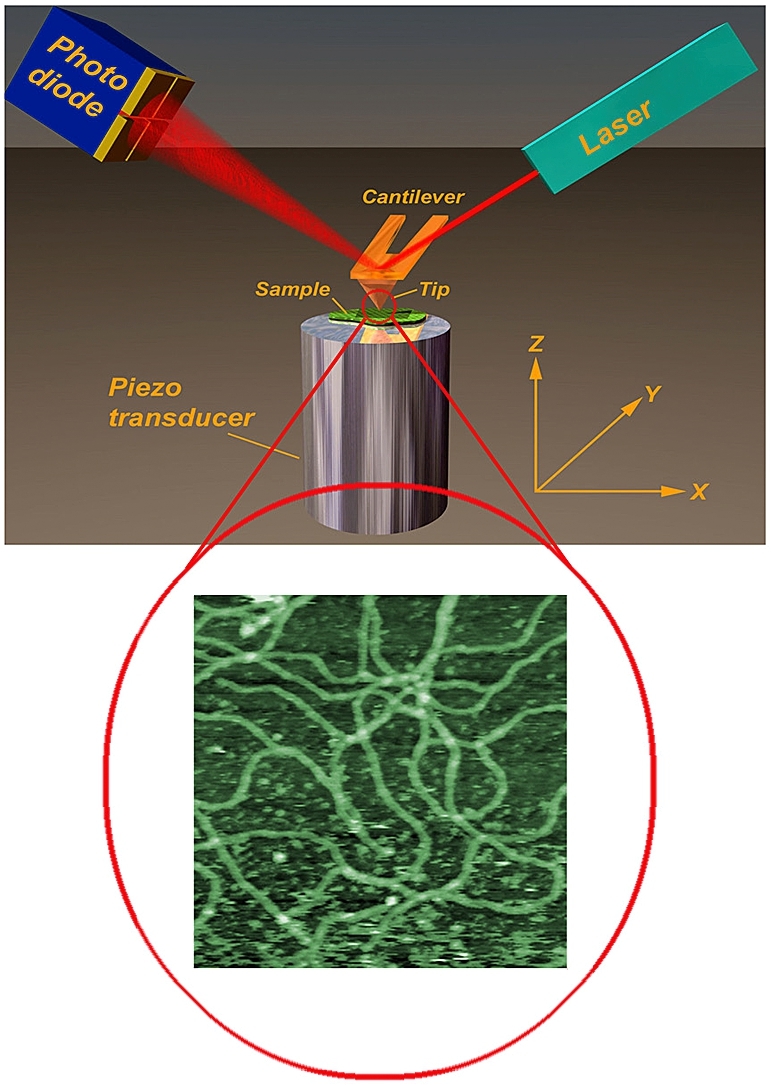

## 1 Introduction

This review is dedicated to Prof. G. O Phillips on the occasion of his 90^th^ birthday. During his extensive research career there has been substantial progress in the understanding of food structure, partly due to the development and application of new experimental methods. Prof Phillips has contributed greatly to the dissemination, development, and application of this knowledge through his own research group, the origination and editorship of the Food Hydrocolloids journal, and the initiation and running of the highly successful Gums and Stabilisers & International Food Colloids meetings. In celebration of this contribution this article reviews developments in the use of one of these new techniques, namely atomic force microscopy (AFM), both as a microscope and as a force transducer. This is a technique we have been intimately involved with over this period. Since its inception in the 1980s AFM has led to considerable insights into a range of food structures. The review will focus on uses in food science and on new developments and, in particular, highlight the increasing use of AFM as a force transducer, which is likely to open up new areas of understanding in the near future.

## 2 Atomic force microscopy – a microscopic tool

An atomic force microscope (AFM) scans a tiny and extremely sharp tip that is mounted on the end of a flexible cantilever over the surface of samples – it is similar to the action of a stylus on a record player, but in terms of microscopy effectively a nano-profilometer. Unlike all other forms of microscopy it has no lenses and does not image the sample by ‘viewing’, rather it does so by ‘feeling’ the surface of the sample (Morris, Kirby, & Gunning, 2009).

### 2.1 Imaging food molecules and structures: early days

The development of atomic force microscopy (AFM) as a tool for imaging biological systems offered considerable promise for imaging at molecular, or sub-molecular level, in a liquid environment (Morris et al., 2009). Realisation of these challenges principally involved the development of an understanding of image contrast in AFM, and development of imaging procedures, which eliminated, or at least minimised, the damage to the sample by the imaging probe. Considerable advances in instrumentation have aided the identification and elimination of artifacts, and there have been major advances in the software produced to run the microscopes and process the images. The AFM offers comparable resolution to that of the transmission electron microscope with the advantage of imaging under natural conditions. This has been achieved for molecules and macromolecular complexes through devising methods for immobilising samples on suitable substrates without destroying or deforming their native structures (Morris et al., 2009). For larger more complex biological systems the challenge is to image the surface, or surfaces of sections of these more complex structures. Through these developments AFM is now becoming used widely to solve problems for biological samples rather than just obtaining good images. Despite widespread use the technique is still not entirely routine and, like other microscopic methods, will always require skill and expertise in modifying sample preparation and imaging methods for particular samples (Morris et al., 2009).

#### 2.1.1 Imaging molecules and complexes: new understanding

**Fig. 1:**
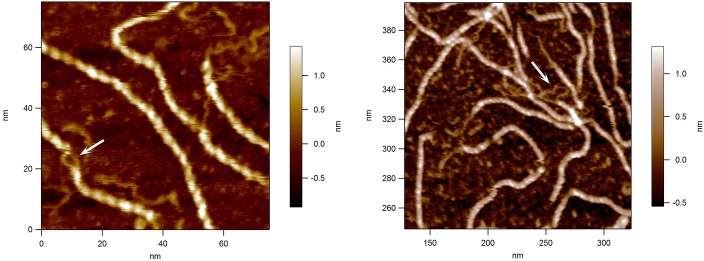
High resolution AFM images of xanthan double helices. The loops at the ends and along the molecule provide evidence for intra- and intermolecular double helix formation. For details see Moffat et al. (2016).

AFM has provided new information on molecular size and shape under more natural conditions. For helix-forming polysaccharides, which function as gelling and thickening agents, it has not only been possible to characterise the molecules but to investigate their functional role. Gellan gum is a model system for investigating polysaccharide gelation, and through the imaging of the molecules, gel pre-cursers and the surface of intact hydrated gels it has been possible to visualise the molecular networks formed in the gels and infer the mode of association (Gunning, Kirby, Ridout, Brownsey, & Morris, 1996). Microgel formation observed in preparations of the thickening agent xanthan gum, provide a basis for understanding the weak gel properties of this material (Morris et al., 2009). Continual progress in instrumentation has, and is still allowing enhanced resolution of molecular structure: in the case of xanthan this has allowed the identification of the arrangement of individual chains in annealed xanthan helices (Fig. 1), providing direct visual evidence for a double, rather than single helical structure (Moffat, Morris, Al-Assaf, & Gunning, 2016).

**Fig. 2:**
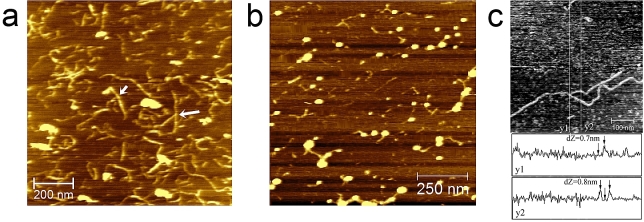
AFM images showing (a) branching of the pectin backbone arrowed, (b) a pectin-protein complex from sugar beet pectin and (c) branching of an amylose molecule. For details see for example Pose et al., 2012, Kirby et al., 2006, Morris et al., 2008 and Gunning et al. (2003).

Microscopic techniques such as AFM allow the characterisation of heterogeneity at the molecular level. An example is the observation of branching in semi-flexible molecules such as arabinogalactans (Adams, Kroon, Williamson, & Morris, 2003). An analysis of AFM images of the binding of inactivated enzymes to arabinogalactans has been used to confirm the random distribution of branches, plus enzymatic creation of non-random distribution of blocks of unsubstituted backbone following enzymatic removal of arabinose branches (Adams, Kroon, Williamson, Gilbert, & Morris, 2004). In the case of pectin extracts AFM revealed (Fig. 2a) irregular branching of the polygalacturonic backbone (Round, Rigby, MacDougall, Ring, & Morris, 2001), which led to a new proposed model for the internal structure of plant cell walls (Vinchen et al., 2003). The enhanced characterisation of pectin extracts has been employed in studies of transgenic strawberry mutants to study the role of ripening enzymes, and has revealed novel structures, previously not seen in cell wall extracts (Pose, Kirby, Mercado, Morris, & Quesada, 2012). AFM showed (Fig. 2b) the nature of pectin – protein complexes (Kirby et al., 2006, Morris et al., 2008), suggested to be responsible for the emulsifying action of sugar beet pectin.

**Fig. 3:**
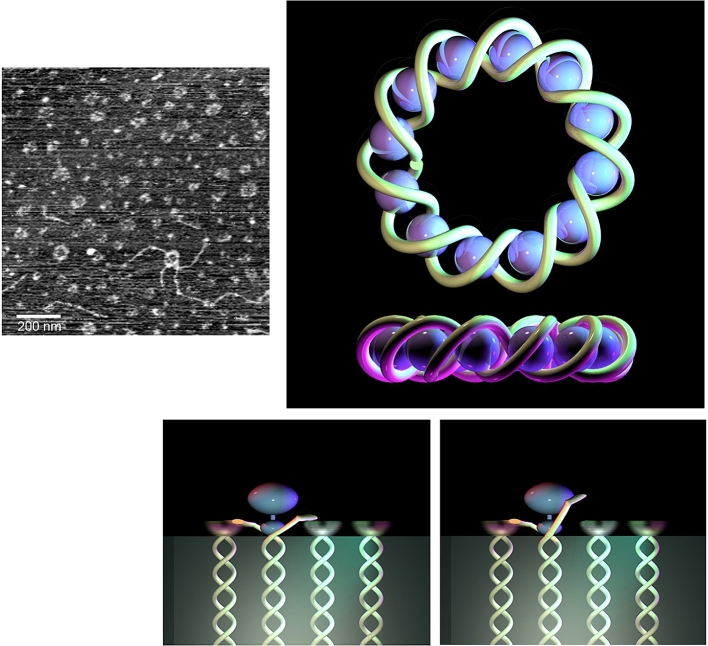
Top left panel, AFM image of SBD – amylose interactions. Schematic diagrams, mechanism proposed for the role of the binding domain in the ability of glucoamylase to degrade crystalline starch (see Morris, Gunning, et al. (2005) and Morris, Ridout, et al. (2005) for further details).

AFM images revealed for the first time irregular low level branching Fig. 2c) of the starch polysaccharide amylose (Gunning et al., 2003). Furthermore, studies of complexes of amylose with mutants of the starch degrading enzyme glucoamylase, revealed how the starch-binding domain (SBD) of the enzyme can bind to amylose helices (Fig. 3). As shown in the AFM image SBDs formed ring-shaped complexes with single amylose chains. Quantification of the chain lengths in the images enabled interpretation of how the rings were formed. Despite the fact that amylose is a polydisperse polymer the distribution of the perimeter length of the rings were half that of the distribution of the contour lengths of the linear chains in all of the images which suggested the amylose chain bound to both binding sites of the SBD. It was known that the binding sites on the SBD are oriented at 90° relative to each other (Sorimachi et al., 1996) and this combination of factors suggested that the SBDs act as substrates for an expanded double helix. This identified the fact that the SBD can recognise, bind and distort the amylose double helix on starch crystal surfaces. This led to a suggested mechanism for the selective degradation of crystalline starch by glucoamylase (Morris et al., 2005).

#### 2.1.2 Imaging molecular assembles: new understanding

The internal structure of more complex biological structures can be investigated by imaging flat cut surfaces or sections of the samples: examples include the study of plant cell walls or starch granule structure (Morris et al., 2009).

In order to investigate the internal structure of starch it was found necessary to embed isolated starch granules in non-penetrating resins, in order to avoid artifacts induced by penetrating resins (Ridout, Gunning, Wilson, Parker, & Morris, 2002), and to cut sections or produce polished flat blocks for imaging (Morris et al., 2005, Ridout et al., 2004). The contrast in the images was shown to be due to different levels of adsorption of water into amorphous and crystalline regions within the granule (Morris et al., 2005, Ridout et al., 2002, Ridout et al., 2004). The subsequent local changes in compressibility and height allowed the distribution of crystalline regions (blocklets) within the granule to be mapped. The images suggested that the widely accepted model of starch, as consisting of alternating crystalline and amorphous rings (growth rings), is over simplistic. The granules were found to consist of blockets (packets of microcrystals arising from the branches of amylopectin structures) arranged radially in an amorphous background. The growth ring structures arise from radial variations in the crystalline/amorphous structure. The imaging methodology was extended to examine the microstructure of self-embedded starch granules in mature seeds (Liu et al., 2013, Parker et al., 2008). The methods developed for imaging starch within seeds allowed the use of AFM, and other microscopic techniques, to be used to define the changes in ultrastructure of starch within starch mutants deficient in specific biosynthetic enzymes, induced as a result of the growth of the seeds (Liu et al., 2013). The important finding was that specific mutations (loss of branching enzyme activity) lead to heterogeneity of ultrastructure, both within and between starch granules in the seeds. The recognition of, and the need to characterise this heterogeneity, is important for understanding the functional properties of high-amylose starches.

The structure and changes in structure of air-water or oil-water interfaces is of crucial importance for understanding the texture, stability and digestion of food foams or emulsions. The use of AFM has led to important new insights into the behaviour of interfaces (Morris & Gunning, 2008).

**Fig. 4:**
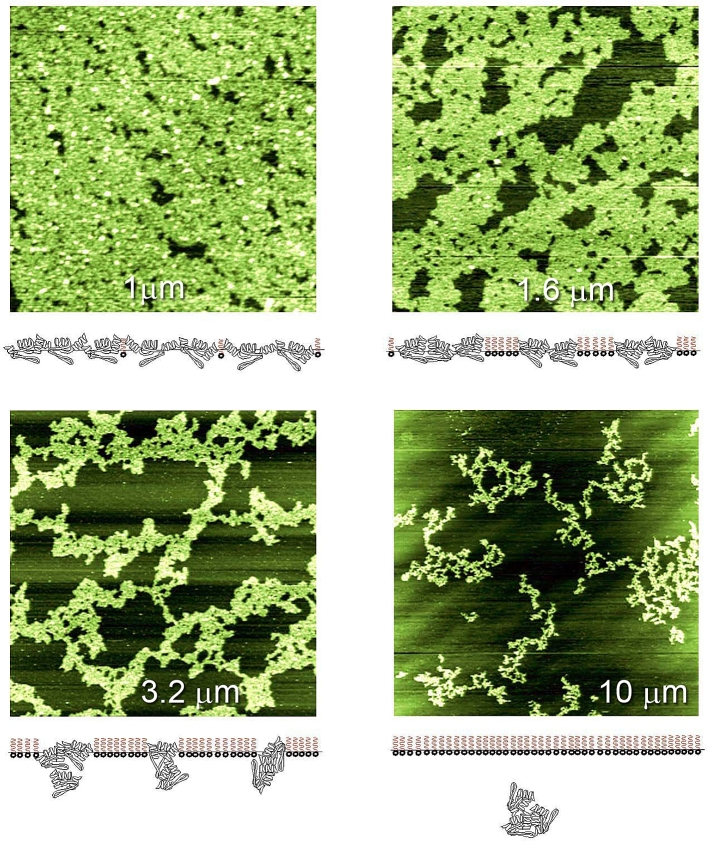
Orogenic displacement of β-lactoglobulin from an air-water interface by the surfactant Tween 20. The images show the growth of surfactant domains, which compress and eventually break the protein network, allowing release of protein into the bulk phase (See Mackie et al. (1999), for further details). Full size of each image is labelled.

AFM proved particularly useful for studying protein networks at interfaces, which were shown to form elastic interfacial networks (Morris & Gunning, 2008). The interfacial structures were sampled using Langmuir-Blodgett techniques and deposited onto flat substrates, such as mica, for imaging (Fig. 4). The earliest experiments were made on air-water interfaces, which were easier to study. AFM images showed that the protein networks were not close-packed structures but were irregular arrays containing holes or defects: a structure consistent with partial unfolding and association yielding an elastic network (Gunning, Wilde et al., 1996). Proteins that form elastic networks at interfaces provide long-term stability for foams and emulsions. Despite this it is possible to destabilise protein-stabilised foams or emulsions using surfactants: AFM imaging discovered the novel mechanism of action (Mackie et al., 1999, Mackie et al., 2000), which was termed the orogenic displacement mechanism illustrated by the schematic diagrams beneath the AFM images (Fig. 4).

Because the proteins are linked into a network it is not possible for the surfactant to displace individual proteins. Rather the surfactant targets the Achilles’ heel of the network – the holes or defects. Increasing surfactant adsorption at the interface leads to growth of surfactant domains (the dark regions in Fig. 4), which compress and eventually break the protein network (the bright regions). Because the AFM monitors the height of the protein network, it proved possible to show that the volume of protein (area x height) remained constant until the network broke, releasing protein. Usually the surfactant is removed prior to imaging the remaining protein network. However, if the surfactant is present it is possible to image the protein displacement as a function of time (Gunning, Mackie, Wilde, & Morris, 1999). Such images show stretching of the protein network leading to breakage and recoil, confirming the elastic character of the network.

The orogenic mechanism proved to be generic, applying to air-water, oil-water interfaces, all surfactants and all proteins studied, which act as foam or emulsion stabilisers (Morris & Gunning, 2008). A measure of the stability of the interface is the surface pressure required to break the protein network. This depends on the protein structure, the nature of the interface and, for oil-water interfaces, the nature of the oil phase, all of which determine the level of unfolding and interaction of the proteins on adsorption (Morris and Gunning, 2008, Maldonado-Valderrama et al., 2010a, Maldonado-Valderrama et al., 2010b). The mechanism also applies to mixed protein networks for which the proteins which, individually form the weakest networks, are preferentially displaced leaving the remaining stronger protein network to determine ultimate failure of the network (Mackie et al., 2001a, Morris and Gunning, 2008). This proved to be important in understanding the behaviour of commercial foam and emulsion stabilisers such as whey protein and sodium caseinate (Woodward et al., 2004, Woodward et al., 2009a, Woodward et al., 2009b). In the case of whey protein the main protein component is β-lactoglobulin, which dominates the behaviour at the interface (Woodward et al., 2004). Sodium caseinate is a commercial stabiliser whose principal components are α- and β- casein, neither of which are themselves, good stabilisers, plus a minor component κ-casein. AFM studies of the individual displacement of α-, β- and κ-caseins showed that the minor component κ-casein was capable of forming a strong network, which failed at a similar surface pressure to that of sodium caseinate: in this case the preferential displacement of the ‘weaker’ proteins (α- and β- casein) effectively concentrated the ‘stronger’ protein, κ-casein, allowing it to form a network which determined the failure of the sodium caseinate network (Woodward, Gunning & Mackie et al., 2009).

**Fig. 5:**
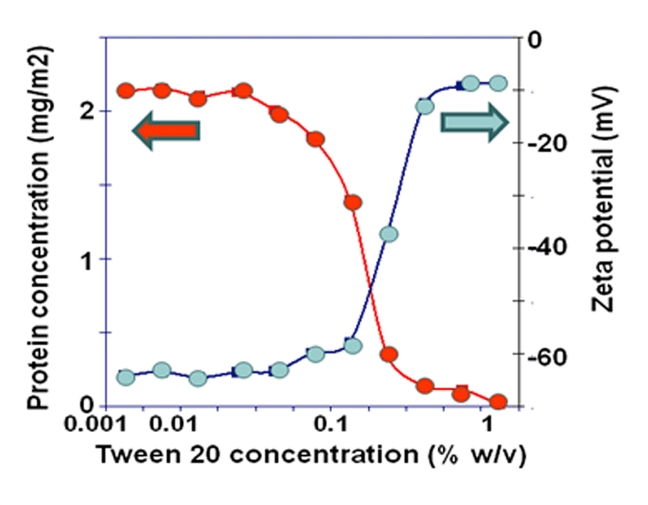
Orogenic displacement of β-lactoglobulin from a tetradecane oil droplet with Tween 20. The protein concentration, monitored with an OPA assay shows the catastrophic release of protein following rupture of the protein network. The uptake of surfactant is shown by the decreasing surface charge on adsorption of the neutral surfactant.

The data described above is based on imaging protein networks transferred to solid substrates. Combined AFM and Brewster Angle Microscopy (BAM) confirmed that the larger surfactant domains observed by AFM, prior to collapse of the protein network, were seen directly at the liquid interface by BAM (Mackie, Gunning, Ridout, Wilde, & Patino, 2001). Studies on liquid lamellae, as models foams, have revealed the presence of highly mobile surfactant and immobile protein phases during displacement (Wilde, Mackie, Husband, Gunning, & Morris, 2004). For emulsion droplets it is possible to monitor the change in droplet deformability corresponding to weakening and rupture of the protein network during surfactant displacement (Gunning, Mackie, Wilde, & Morris, 2004) or, as shown in Fig. 5, make direct measurement of surface protein concentration with respect to the change in surface charge accompanying surfactant adsorption.

An understanding of protein-surfactant interactions at interfaces provides a basis for improving, rationally the stability of the interface, and hence the lifetime of the product. However, the methodology can be extended to investigate the changes in interfacial structure during digestion. Experimental studies on the effect of digestion conditions on interfacial structures were initially made on model air-water systems, and then extended to oil-water interfaces as more realistic models of food emulsions.

If a droplet from a processed food emulsion with an intact protein network survives transit through the stomach then bile salts will attempt to displace the protein. The level of displacement is important since the bile salts act as sites for the localisation of lipase-colipase complexes and their surface concentration will determine the rate of lipolysis. AFM studies under *in vitro* duodenum conditions show that displacement occurs via an orogenic mechanism (Maldonado-Valderrama et al., 2008). Thus if the total area occupied by bile salts could be controlled by the strength of the protein network, or if the protein network could be strengthened to reduce the surface concentration, then this offers a route to reducing the rate of fat/lipid hydrolysis, and hence potentially induce physiological benefits such as reduced or moderated fat intake and satiety. *In vitro* studies have shown that this approach was feasible (Woodward, Gunning & Wilde et al., 2009). To assess this approach it is necessary to define the effects of gastric conditions on interfacial protein networks to establish how such structures are affected by passage through the stomach.

Effects of gastric conditions on interfacial protein networks were studied for air-water interfaces and then extended to more realistic oil-water systems. At air-water interfaces AFM together with surface tension and interfacial rheology measurements were used to assess the effect of gastric conditions on pre-formed interfacial β-lactoglobulin networks. Individual changes in conditions (acid pH, ionic strength, body temperature) generated small changes in structure but, unexpectedly combined effects of pH and temperature weakened the networks which, never-the-less, still remained intact. For oil-water interfaces the effects of gastric media on pre-formed β-lactoglobulin layers adsorbed at two different oils (tetradecane and olive oil)-water interfaces showed the importance of the nature of the oil phase on network stability and that protein unfolding, induced by the oil phase, may offset certain aspects of the weakening of the networks induced under gastric conditions (Maldonado-Valderrama et al., 2010b, Maldonado-Valderrama et al., 2009).

Pepsin was used to investigate exposure of interfaces to proteolysis in the stomach. Understanding protein unfolding on adsorption and under gastric conditions proved important. At air-water interfaces the action of pepsin under gastric conditions on pre-formed β-lactoglobulin networks led to partial hydrolysis of all surface-adsorbed proteins: however, proteolysis did not disrupt the protein network which remained intact and capable of resisting surfactant displacement. Surfactants may be present under gastric conditions and, since orogenic displacement causes enhanced exposure of protein structure to the aqueous media, their effects on proteolysis were studied. Modification of the surface conformation of the proteins during ‘orogenic’ displacement led to an unexpected synergism, which enhanced proteolysis. However, importantly, strengthening of protein networks to inhibit surfactant domain growth should thus not only restrict bile salt adsorption, but also inhibit proteolysis in the stomach. Such observations are important new generic features of digestion, which can be manipulated for rational design of food structures to promote health.

Studies at air-water interfaces were extended to oil-water interfaces and emulsions. The generic features were retained at the oil-water interfaces and the nature of the oil phase is of importance. Studies at interfaces were extended to study effects of gastric digestion on protein-stabilised oil-water emulsions: linking basic studies to realistic models. Interestingly, digestibility profiles of interfacial proteins depended on the nature of the oil phase. The type of oil affects the surface conformation of the protein affecting proteolysis in the stomach. Proteomic analysis of the peptides generated during the digestion process provide new information on proteolysis by pepsin under gastric conditions, as well as novel information about the interfacial properties and conformation of β-lactoglobulin adsorbed at different oil-water interfaces (Maldonado-Valderrama et al., 2012, Woodward et al., 2010).

In general polysaccharides are poor emulsifiers. However, certain protein-polysaccharide complexes, such as gum Arabic and sugar beet pectin, show emulsifying properties, attributable to the protein component. In the case of sugar beet pectin AFM studies provided insights into the role of the protein. AFM images (Fig. 2b) of sugar beet pectin extracts showed that about 60% of the pectin molecules were present as pectin-protein complexes (Kirby et al., 2006). In emulsions the protein component was proposed to adsorb at the oil-water interface with the polysaccharide component extending into the aqueous phase surrounding the oil droplets. It was suggested that the polysaccharide layer extended into the water phase causing steric repulsion between droplets and inhibiting coalescence (Leroux, Langendorff, Schick, Vaishnav, & Mazoyer, 2003).

**Fig. 6:**
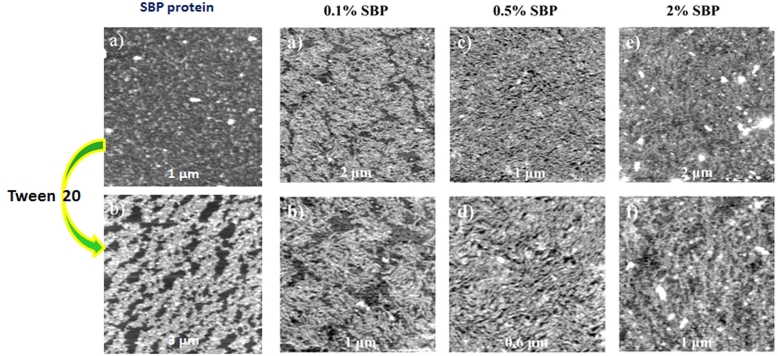
Showing interfacial structures formed by sugar beet pectin. Full size of each image is labelled. Cleavage of the complexes showed that the protein alone forms elastic networks (see displacement with Tween 20). Pectin in the sugar beet pectin complexes (SBP) chains protect and strengthen the protein network, and the extension of the polysaccharide layer into the bulk phase increased with the bulk SBP concentration. (For details see Gromer et al., 2009).

In order to test this model for emulsification AFM was used to image the structures formed at interfaces (Fig. 6), and then force spectroscopy was used to study the effects of these interfacial structures on the interactions between oil droplets in an aqueous medium (Gromer et al., 2009, Gromer et al., 2010). Initially studies were made of the structures formed at air-water interfaces, which are easier to image, and then extended to view structures formed at oil-water interfaces. The AFM studies were supported by measurements of interfacial tension and rheology. Cleavage of the complexes resulted in the formation of elastic protein interfacial networks as evidenced by their orogenic displacement with Tween 20. At air-water interfaces the complexes formed protein network containing holes (defects) and protected and strengthened by ‘rod-like’ pectin chains. Similar structures were formed at oil (tetradecane)-water interfaces. The complexes formed protein networks containing holes (defects) and protected and strengthened by ‘rod-like’ pectin chains. As the bulk concentration of the SBP extract increased, the size of the defects in the network decreased and the surface became rougher with increasing extension of the pectin chains away from the interface (Gromer et al., 2009). The effect of these interfacial structures on droplet-droplet interactions was probed by force spectroscopy (Gromer, Penfold, Gunning, Kirby, & Morris, 2010) as discussed in section 3.1.2.

## 3 Atomic force microscopy – a force transducer

### 3.1 Force measurements: early days

A useful aspect of AFM compared to every other form of microscopy is that in addition to imaging it can measure force. The force measurement by AFM is actually a form of spectroscopy, which means it gathers several factors; force magnitudes, distances and timescales which enables many forms of investigation. Factors such as the mechanical modulus of the sample can be determined through indentation of the AFM tips onto samples (Calabri, Pugno, Menozzi, & Valeri, 2008) and, in addition, adhesion between the tip and sample can be quantified by recording the motion of the AFM tip and cantilever upon retraction from the sample surface. Retraction applications include fundamental elastic properties of synthetic polymers (Giannotti & Vancso, 2007), the effects of primary and secondary structure on the mechanical properties of proteins (Ng, Randles, & Clarke, 2007), double and single stranded nucleic acids (Strunz, Oroszlan, Schäfer, & Güntherodt, 1999), and polysaccharides (Marszalek et al., 1998, Marszalek et al., 2001). The stretching of semi-flexible polymers is described by two statistical models, the Kratky-Porod worm-like chain model (WLC) (Flory, 1998, Kratky and Porod, 1949) and the freely-jointed chain (FJC) (Beuche, 1962, Smith et al., 1996) model. These models are equivalent but use different concepts to describe stiff chains. The WLC model introduces the concept of a persistence length l_P_. The most commonly applied formula describes the extension, z, of a worm-like chain with contour length L_c_ and persistence length, l_P_, in response to a stretching force, F, as;

$$F\left(z\right)=\frac{k_{B}T}{l_{P}}\left[\frac{1}{4}{\left(1-\frac{z}{L_{c}}\right)}^{-2}+\frac{z}{L_{c}}-\frac{1}{4}\right]$$

K_B_, is Boltzmann’s constant and T, absolute temperature (Bustamante et al., 1994, Marko and Siggia, 1995).

In the FJC model stiffness is described by effectively replacing the monomer lengths by Kuhn statistical segment lengths, l_K_ connected by flexible linkages, in order to account for the increased volume. For semi-flexible polymers the Kuhn length is twice the persistence length. The FJC model is particularly suited for describing polymers lacking secondary structure but showing restricted rotation of the monomeric units about the inter-monomer linkages. The general form is given in this equation below:

$$Z\left(F\right)=L_{C}\left[\coth\left(\frac{Fl_{K}}{K_{B}T}\right)-\frac{K_{B}T}{Fl_{K}}\right]$$

To extend this model to the higher force ranges more commonly employed in force spectroscopy experiments of ordered stiffer polymers such as DNA, a modification was developed which imbues the segments with a finite elasticity so that the chain is treated as a series of springs (Smith et al., 1996):

$$Z\left(F\right)=L_{C}\left[\coth\left(\frac{Fl_{K}}{K_{B}T}\right)-\frac{K_{B}T}{Fl_{K}}\right]\left(1+\frac{F}{K_{S}l_{K}}\right)$$

l_k_ is Kuhn length and, K_S_ an elasticity parameter.

Another aspect of retraction force measurement is exploration of ligand-receptor interactions by functionalising the AFM tips (covalent attachment of specific molecules). Tip functionalisation protocol includes use of a heterobifunctional spacer molecule, esterified polyethylene glycol (PEG) (Hinterdorfer et al., 1996, Hinterdorfer et al., 2002). It has reactive covalent esters at each end, which enables the ligand molecules to be attached to the AFM tip in a manner like that of a baited hook on the end of a fishing line. It is a better method than attachment of ligand molecules directly to the surface of the AFM tip as the PEG spacer allows them to be rotationally mobile so they can ‘dock’ successfully with their target receptor. Force - distance cycles are carried out by using the functionalised AFM tip to fish for the complementary ligand or receptor on a sample surface and measuring the magnitude and frequency of adhesive interactions seen upon retraction of the tip. Once tip-sample binding has been established in a repeatable manner, the specificity of the interaction can be unequivocally established by adding the complementary partner for the active species, or a suitable inhibitor, as a free molecule into the solution. AFM force spectroscopy of ligand-receptor interactions has been mathematically modelled (Evans and Ritchie, 2007, Strunz et al., 2000) to enable higher precision of the molecular interaction to be gathered rather than through use of the traditional techniques such as microarrays. In addition to quantification of ligand-receptor binding strength the models calculate the off-rate constant of dissociation, and the number and length of energy barriers.

Force–distance curves have been successfully modelled in a recent ground-breaking study that demonstrated that deformable colloids such as oil droplets and air bubbles are particularly susceptible to studies by AFM because they adapt their shapes in response to the changing forces caused by the non-adsorbed species (which can also include surfactant micelles) in the closing gap (Tabor, Chan, Grieser, & Dagastine, 2011). This opens up the potential for tailoring colloidal interactions within complex systems in a properly knowledge-based way for the first time.

#### 3.1.1 Force measurements on molecular complexes

The potential bioactivity of polysaccharides has been recognized for many years, particularly in the area of dietary fibre and the beneficial effects this material has on gut health (Bingham et al., 2003). Recent research has shown that certain polysaccharides can exhibit anti-cancer properties (Nangia-Makker et al., 2002, Olano-Martin et al., 2003) but there is a need to establish molecular mechanisms. To investigate the reported anticancer properties of modified pectin, the interaction between defined fragments of pectin molecules and the important tumour signalling molecule Galectin-3 [Gal-3] were measured by force spectroscopy (Gunning, Bongaerts, & Morris, 2009). Gal-3 is a galactose binding lectin, and the suggested hypothesis for the anti-cancer action of pectin is that components of chemically- or enzymatically-modified pectin extracts binds to and inhibits the biological activity of the Gal-3 (Kidd, 1996) by preventing its association with natural receptors (Sundblad, Croci, & Rabinovich, 2011). The force spectroscopy data obtained showed specific binding between pectin fragments containing available linear galactan chains and the carbohydrate binding domain of Gal-3. Further studies confirmed specific binding of galactobiose to Gal-3 (Gunning, Pin, & Morris, 2013) and characterised the nature of the binding. These studies used commercially available samples and their structures were checked by nmr spectroscopy: The galactan regions in the pectin samples that bound specifically to Gal-3 were found to be pure β(1–4) linked galactans in this study.

**Fig. 7:**
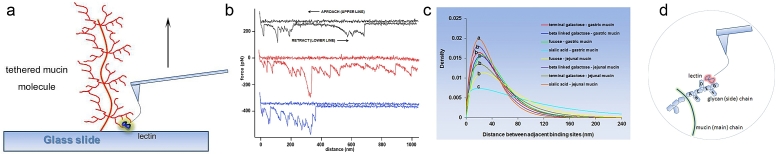
AFM measurement of the glycan distribution on mucin. (a) Schematic diagram of the technique. (b) Three examples of force spectra data. (c) Mathematically modelled fitting of the measured glycan distributions in two different mucin phenotypes. (d) Schematic diagram of antennae composition.

One of the main molecules which protects the gastrointestinal tract is mucin; a glycoprotein composed of a polypeptide backbone with a very large number of glycan sidechains. The mucin molecular mass is generally composed of >50% and often 70–80% carbohydrate. Mucin is a highly heterogeneous polymer in terms of its glycan sidechains (Robbe, Capon, Coddeville, & Michalski, 2004). The sidechains are multiply branched and named as antennae, giving the mucin a bottle-brush appearance. The significant aspect of the carbohydrate presence in mucin is that sugars can encode dramatically more bio-information, due to their significantly higher variation in structural arrangement compared to the other bio-encoding molecules such as DNA and proteins (Davis, 2000, pp. 134–138). Sugar molecules have available a wider variety of linkages than DNA bases and amino acids, and in addition the spatial orientation of their hydroxyl groups can vary and also have offer numerous substitution sites. The effect that the structural variation of carbohydrates has on biomolecular interactions involving signalling is termed the ‘glycocode’. AFM force spectroscopy can be used to characterise mucin at molecular resolution (Gunning, Kirby & Fuell et al., 2013). The AFM tips are functionalised with the specific carbohydrate binding proteins, lectins (Iskratsch, Braun, Paschinger, & Wilson, 2009), that interact with the sugar components of the mucin antennae (Fig. 7a). The force spectroscopy data obtained (Fig. 7b) can be mathematically modelled to characterise the distribution of the specific carbohydrate species and reveal differences in the highly complex structures of mucins (Fig. 7c). Fitting the adhesion event distances from the range of the specific binding lectins allows quantitative comparison of the different glycan epitopes present within a given mucin, which reveals detail of the structural composition of the antennae (Fig. 7d).

Despite the inherent heterogeneity of the glycan substitution on mucins, the fact that this technique was able to discriminate mucins from different regions of the gut and track changes induced by enzymatic attack holds much promise. AFM can, not only begin to read the glycocode on mucin, but also investigate which external factors (dietary components such as pre-biotics and pro-biotics) may re-write it.

#### 3.1.2 Force measurements on molecular assemblies

A new technique for direct examination of colloidal interactions was provided by AFM (Butt, 1994, Ducker and Senden, 1992). This form of microscopy can measure forces at levels from single molecular interactions up to deformation of very soft and very hard materials (Calabri et al., 2008), which has made a significant impact in physics, chemistry and biology. The important aspect of this, in terms of food material science, is that all three of these science areas are encountered.

**Fig. 8:**
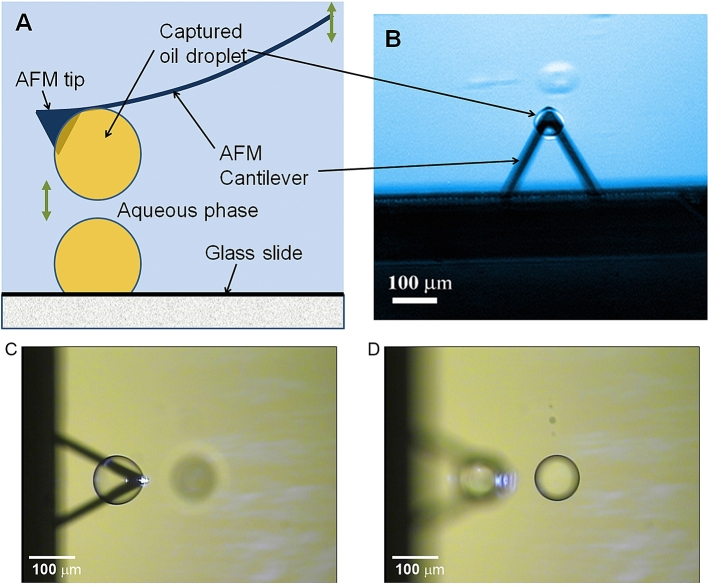
(A) Oil droplet capture scheme (B, C) optical images of attached droplets on the AFM cantilevers, and (D) on the glass slide.

Fig. 8 illustrates how droplet interactions are measured by force spectroscopy. The droplets are attached to the end of the AFM cantilever by pressing the tip into an oil droplet sitting on the glass slide in water and then pulled away from the surface of the slide. Fig. 8D shows the focused image of the droplet on the glass slide. The fortunate factor is that the spraying deposition process of oil droplets onto glass slides (Gunning et al., 2004) created many equally sized droplets, which enabled proper alignment of matching pairs for the force measurements to be carried out. The initial stages of measuring colloidal interactions with AFM was carried out with solid particles attached to the cantilevers pressed against large bubbles (a few hundred microns) attached to the substrate (Ducker & Senden, 1992; Butt 1994) but the later studies revealed the significant differences in colloidal interactions between pairs of deformable particles such as oil droplets (Dagastine et al., 2004, Gunning et al., 2004) and air bubbles which were attached to the cantilevers as well as the substrate (Tabor et al., 2012, Vakarelski et al., 2008). In addition to high deformability the interfacial films can also be mobile in liquid and gas colloids, which can vary the local interaction region.

**Fig. 9:**
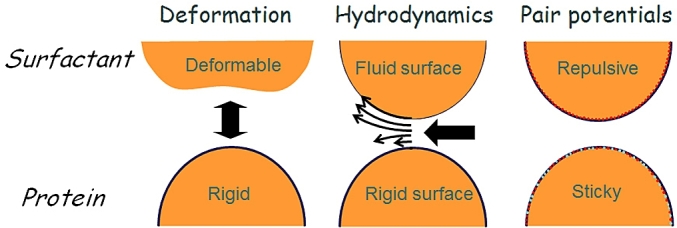
Characterisation of surfactant and protein emulsified droplets.

Oil droplets can be emulsified by two different compounds; surfactants and proteins. As illustrated in Fig. 9 the stabilisation methods of each are different. Surfactants create a fluidic mobile homogeneous interface with lower interfacial tension. Proteins create an elastic rigid interface with heterogeneous distribution of charge and hydrophobicity. These major differences provided an incentive for investigating how they may alter the force interactions between oil droplets using AFM. As droplets approach each other the flow of the liquid across the surface of the droplets can vary depending upon the mechanical nature of the interfacial region as shown by the length of the arrows (Fig. 9). Previous interfacial AFM studies demonstrated the details of how interfacial protein films can be displaced by surfactants (Mackie et al., 1999, Mackie et al., 2000) and this prompted force spectroscopy measurements on a pair of oil droplets stabilised with an interfacial protein film which was then displaced by surfactant (Gunning et al., 2004). An interesting discovery was that the deformability of the oil droplets when coated with protein showed unexpected behaviour. The internal pressure (P) within the droplet should follow Laplace’s law:

(1) $$\Delta P=\frac{2\gamma }{r}$$

where *γ* is the surface tension and *r* the droplet radius. Thus adsorption of protein should lower the surface tension, lowering the internal pressure making the droplets more deformable.

**Fig. 10:**
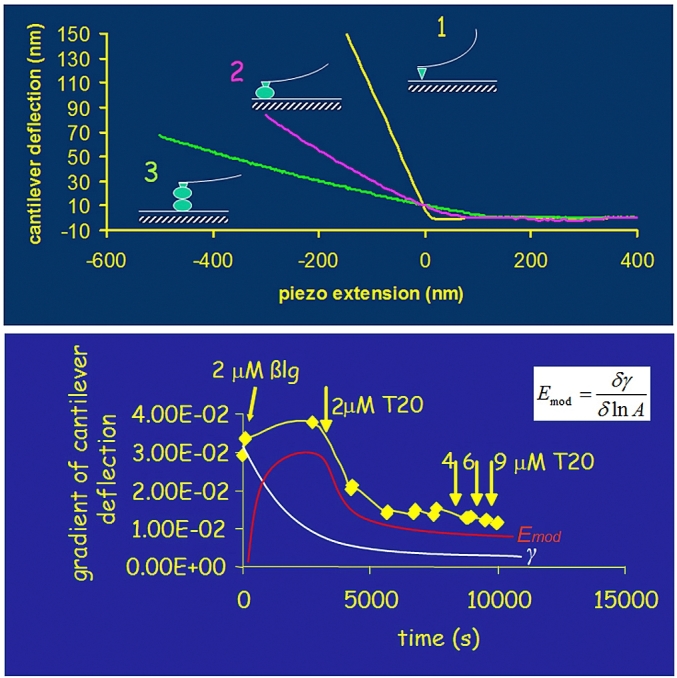
(Top panel) Plots of cantilever deflection versus distance: (1) no oil droplet (2) one oil droplet (3) two oil droplets. (Bottom panel) Evidence that droplet deformability doesn’t simply follow interfacial tension trend (white line) but matches the elastic moduli (red line). For details see Gunning et al. (2004).

The measurement of deformability of soft materials by AFM is quantified by the variation of the deflection of the cantilever as the sample is pressed against a rigid surface. The deformation of a soft sample will reduce the deflection of the cantilever compared to that seen for the cantilever alone. Fig. 10 (top panel) shows an example of the reduction in cantilever deflection reflecting the level of deformation of the material. Curve 1 is for the AFM tip alone pressed against a glass slide, both of which are totally rigid hence the gradient value is 1.0 as the cantilever moves precisely the same distance as the z piezo extension of the scanner, which is pushing them together. When a tetradecane droplet was added to the end of the cantilever and pressed against the glass the deflection gradient dropped to 0.5 as the droplet is deformable and the cantilever only moves 50% of the distance of the piezo scanner. When the tip-attached droplet was then pressed against a sessile droplet on the slide the gradient fell even lower, to 0.25. It shows that the gradient value reflects mechanical properties of the deformable materials (i.e. the droplets act like addition of springs to the cantilever). The yellow line in Fig. 10 (bottom panel) shows changes in the measured gradient of the gradient as a pair of droplets were pushed together. Initially the uncoated tetradecane droplet was studied. Then an amphiphilic protein, β-lactoglobulin (βlg), was added to the bulk water phase and left to self-assemble into an interfacial protein film on the droplets. The plot of the cantilever deflection gradient versus time shows that the measured gradient increased despite the reduction in interfacial tension (white line), which should lower the droplet internal pressure. Then 2 μM of non-ionic surfactant Tween-20 (T20) was added to the water phase to displace the interfacial protein from the droplets to allowing direct comparison of the droplets interaction once coated with surfactant. The cantilever gradient value dropped with time as the surfactant displaced the protein. In order to interpret the surprising variation in droplet deformability an independent measure of interfacial tension (white line) and elastic modulus (red line) was carried out by Pendant drop analysis of a tetradecane droplets in water for the same concentrations and timescales of the addition of βlg and then T20. This showed that the dominant factor in the deformability of the protein-coated droplets was the increasing elastic moduli of the interfacial protein film, not the reduction of interfacial tension. This provided confirmation that protein emulsified droplets become more rigid due to their interfacial ‘coats’ unlike the behaviour of surfactant emulsified droplets, which become more deformable.

**Fig. 11:**
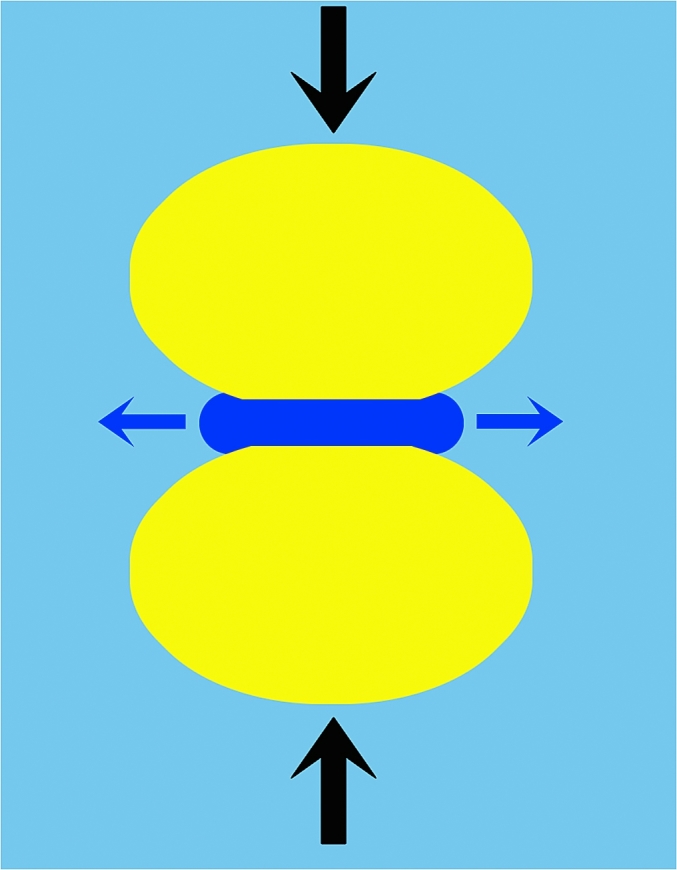
Schematic of the hydrodynamic flow of the thin liquid film (dark blue) of the bulk phase (light blue) that is squeezed out as the droplets (yellow) are pushed together. (For interpretation of the references to colour in this figure legend, the reader is referred to the web version of this article.)

As droplets collide the nature of the interface will determine the flow of liquid from between the converging droplets. Force spectroscopy can be used to monitor differences in the hydrodynamic flow of the thin liquid film (TLF) that becomes forced out as the droplets are pushed together as illustrated in Fig. 11 (Gunning Kirby & Wilde et al., 2013).

Force spectroscopy of droplet-droplet interactions were obtained by positioning the cantilever-droplet assembly directly over another droplet of approximately equal size, which was bound to the glass slide. Each measurement consisted of a force versus distance cycle in which the droplets (initially separated) were first pushed together, and then pulled apart. Relaxation processes were captured by imposing a fixed dwell time following the approach part of the force versus distance cycle, once the cantilever deflection had reached a predetermined value of loading force, termed ‘trigger force’. During the dwell period the feedback loop of the AFM holds the z stage of the piezoelectric scanner in position (using a second feedback loop to eliminate any piezoelectric creep) and deflection of the cantilever is monitored with time.

**Fig. 12:**
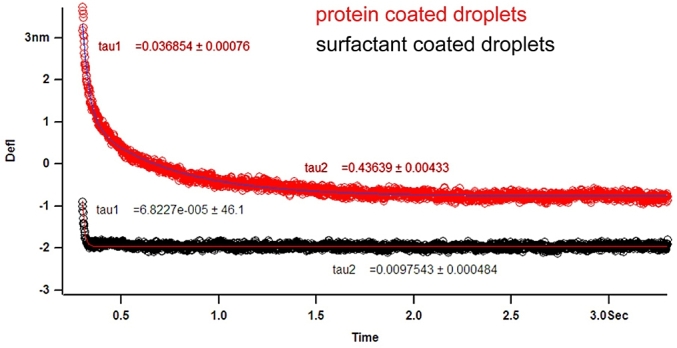
Thin film hydrodynamic drainage relaxation data double exponential fitted to quantify the variation between protein and surfactant emulsified droplet interactions (for details see Gunning et al., 2013b).

**Fig. 13:**
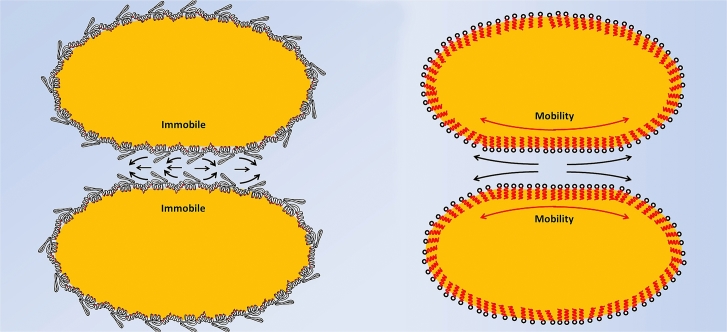
Schematic diagram of thin liquid film flow rates in protein (left) and surfactant (right) emulsified droplets.

The experiments provided direct measurement of the drainage of the thin liquid film. The data in Fig. 12 reveals the significant difference between protein-coated and surfactant-coated droplets. The fact that the interfacial protein film is immobile did dramatically reduce the hydrodynamic flow, which creates a significant change in the droplet interactions (Fig. 13).

**Fig. 14:**
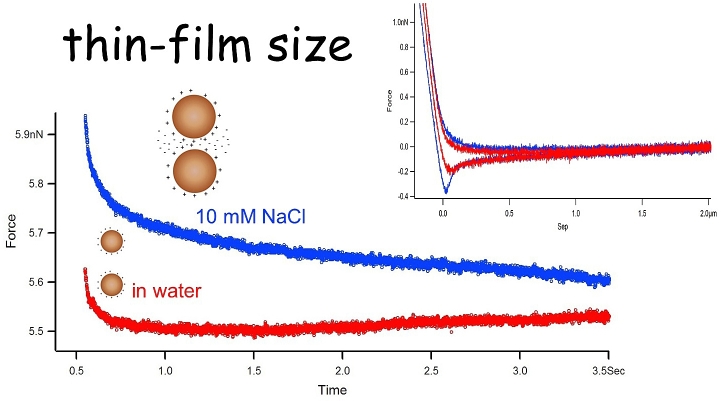
Effect of electrostatic screening on the separation between approaching droplets. Inset: the complimentary force distance curves.

The hydrophilic component of the protein in the aqueous phase can slow the flow of the water molecules as the droplets are pushed together but more data in the study revealed that the flow rate slows further as they become closer (Gunning Kirby & Wilde et al., 2013). Two factors affected this behaviour: (i) having a higher loading force setpoint (trigger) and (ii) increasing the ionic strength of the aqueous bulk phase with screening counterions which lowered the Debye length. Hence the thin liquid film became thinner as the droplets were pushed together even at lower forces. Although droplet separation could not be measured easily in these experiments, one can manipulate the ionic strength of the continuous phase in order to exert control over the thickness of the aqueous films that form between approaching droplets. If the experiment is carried out in pure water, the screening of the electrostatic charges present on the droplets will be very low, and the Debye layers associated with each droplet will overlap at relatively large separation distances, causing repulsion at large separations. Due to their deformability the distance of closest approach for droplet surfaces is controlled by the magnitude of the repulsive disjoining pressure (Carnie, Chan, Lewis, Manica, & Dagastine, 2005). Once the disjoining pressure reaches the strength of their deformability the droplets will begin to form a flattened face, as shown in Fig. 11, that grows radially as they approach each another (i.e. the aqueous film between the droplets will not thin any further in response to squeezing). If salt (NaCl) is added to the bulk phase, the Debye layers are compressed, allowing the droplet surfaces to get closer together before they feel any electrostatic repulsion, leading to much thinner aqueous films between the droplets. Fig. 14 shows relaxation curves obtained for a pair of β-lactoglobulin-coated droplets in pure water, and the data obtained for the same droplets following the addition of salt to the liquid cell. At low ionic strength (water) the relaxation is relatively fast, and the data resembles the trend seen in the data for surfactant-coated droplets. Following the addition of salt the relaxation becomes markedly slower and larger in magnitude.

**Fig. 15:**
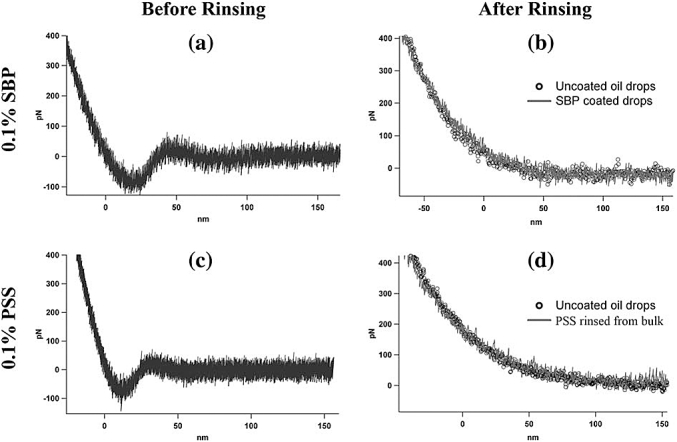
Effect of polymers on oil droplet interactions. (a,c). The force–distance curves were acquired in the presence of the polymer solution (a and c) and after rinsing the bulk aqueous phase with pure water (b and d). The shape of the force–distance curve obtained in polymer solution is compatible with a depletion interaction. In these spectra, the approach and retract curves are superposed. In (a), the mean adhesion is: 142 pN 14, based on 19 curves (RMS noise: 70 pN), and in (c), the mean adhesion is: 134 pN 18, based on 15 curves (RMS noise: 78 pN). For both SBP and PSS the interaction observed in polymer solution disappeared when the bulk aqueous phase was rinsed with pure water and the resulting spectra (lines) are similar to those for uncoated oil drops in water (open circles). For the sake of clarity, (b) and (d) only show the approach curves: in this case the approach and retract curves are completely superimposed. Copy of Fig. 3 from Gromer et al., 2010.

The force spectroscopy measurements can be used to extend studies of droplet-droplet interaction for systems where the interfacial structure is well defined. An example is the case of droplets stabilised with sugar beet pectin (SBP) extracts (Gromer et al., 2010). Sugar beet pectin acts as an emulsifier (Leroux et al., 2003; Williams et al., 2005; Funami et al., 2007). AFM imaging showed the presence of protein-polysaccharide complexes (tadpoles) in sugar beet pectin extracts (Kirby et al., 2006, Kirby et al., 2008). Imaging of the interfacial films formed by sugar beet pectin extracts (Gromer et al., 2009) revealed an elastic protein network screened by the attached polysaccharide. At low bulk phase concentrations the adsorbed SBP interfacial layer was relatively flat (Fig. 6). Force spectroscopy revealed depletion interactions between the droplets in the presence of non-adsorbed SBP in the bulk phase (Fig. 15). Depletion is caused by the osmotic pressure exerted when non-adsorbing polymers are squeezed out of the thin aqueous film that exists between two close-packed colloidal particles (Asakura & Oosawa, 1954). This was confirmed by two methods: (i) replacing the bulk aqueous phase with pure water, which removed the oscillatory sections in the force curves (right panels, Fig. 15b). (ii) measuring interactions between oil droplets in an aqueous bulk phase that had a non-absorbable polyelectrolyte polymer, polystyrene sulphonate (PSS), which also showed the same oscillatory effects on the force curves and elimination of the effect when the bulk polymer was removed (Fig. 15 c,d).

**Fig. 16:**
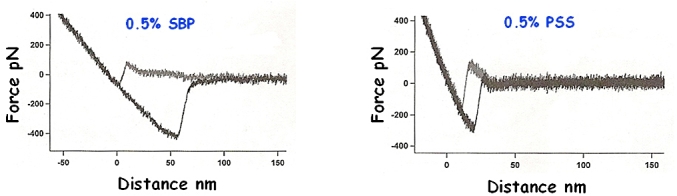
Effect of higher concentrations of the polymers on oil droplet interactions. Grey – approach, black – retract.

Higher concentrations of each of the polymers in the bulk phase (0.5%) caused hysteresis between the approach and retract phases (Fig. 16). This revealed that there is a strong liquid structural correlation occurring within the liquid film separating the droplets. At this higher polymer concentration jump-in features always appeared on the approach part of the force curves and also the subsequent adhesive peak upon droplet separation in the retract curves.

**Fig. 17:**
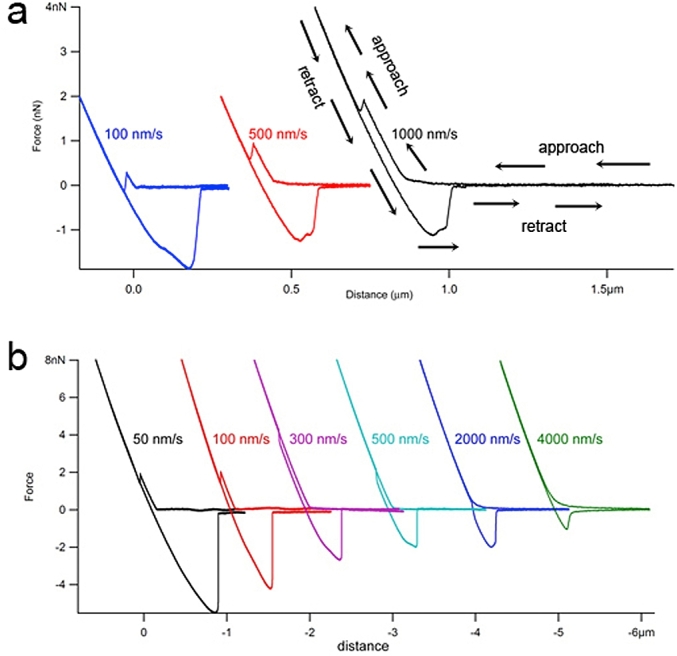
Droplet interaction data in presence of (a) 2% polystyrene sulphonate (b) 3% polystyrene sulphonate. Note an arbitrary distance offset has been added to separate the data sets.

Further confirmation that it was due to the structural correlation of the liquid film separating the droplets was provided by varying the polymer concentrations and also the velocity of the droplets being pushed together and pulled apart (Fig. 17). The depletion effects between droplets were explored further using the non-adsorbing polyelectrolyte polymer, polystyrene sulphonate (PSS). This enabled measurements at higher polymer concentrations without masking depletion effects of the steric repulsion seen in SBP. The relatively low molecular weight and random coil nature of PSS means that it does not affect the viscosity of the continuous phase as much as occurs for the pectin. The jump-in features and an adhesive peaks upon droplet separation in the retract curves revealed two interesting effects. The position of the jump-in moves progressively up the approach curve with increasing approach speed whilst at the same time the magnitude of the adhesion peak reduces.

**Fig. 18:**
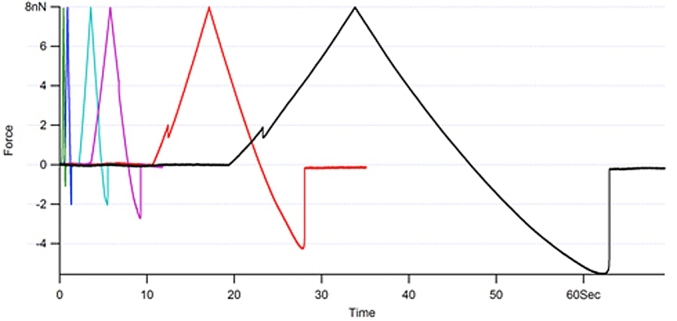
Chronology of droplet interaction data in presence of 3% polystyrene sulphonate.

As the PSS concentration was increased further the effects became more pronounced (Fig. 17b) and the jump-in moves all the way onto the retract portion of the data. This may appear counter-intuitive but is completely unique to deformable colloid interactions. As they are pushed together the deformation stores elastic energy. As the piezo scanner moves into retract phase the force on the cantilever drops, hence the curve turns through 180° but the thin aqueous liquid film trapped at the point of contact between the droplets will continue to be squeezed, and therefore thin-out for some time even as they are pulled apart, explaining how jump-in events can occur even during the retraction part of the force-distance curve. This unique effect on deformable particles has been confirmed by theoretical modelling (Gromer et al., 2010). Examination of the data reveals that there is a correlation between the position of the jump-in and the magnitude of the adhesion when the droplets are finally pulled apart. Fig. 18 shows a plot of the force data against time, which makes the correlation more obvious.

**Fig. 19:**
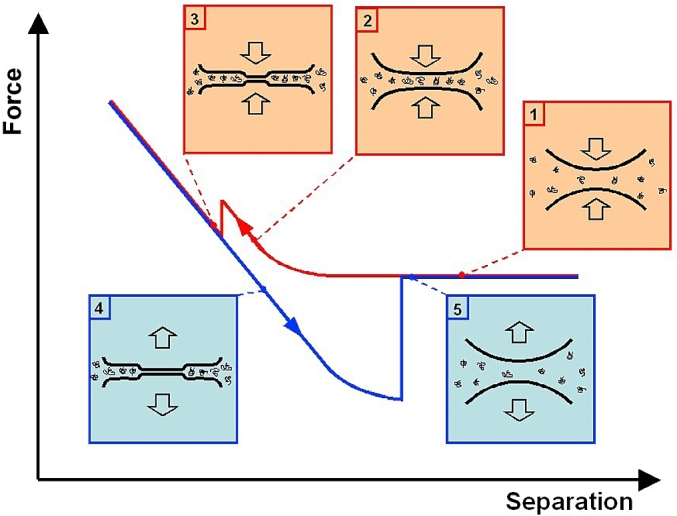
Interpretation of the hysteresis effect observed in the interaction between oil droplets inside a polymer solution [1]. When the droplet surfaces are far apart they don’t interact [2]. As the droplet surfaces come sufficiently close together, they start to deform; polymers remain in the thin liquid film between them [3]. As the polymers diffuse away from a region of the film, a ‘snap in’ effect occurs which corresponds to the formation of a black spot, i.e. the local lamella thickness has reached the dimension of common black films [4]. The black spot expands with time and it keeps expanding even as the droplets are pulled apart, until a ‘snap out’ is observed which corresponds to the droplets being suddenly disconnected [5]. After the droplets have been separated, the droplet surfaces recover their initial shape.

The longer the time duration following jump-in, the larger is the subsequent pull-off required to separate the droplets. The interpretation of these effects is that they result from the formation of a region devoid of polymers in the thin liquid film between the drops (an analogue of this effect is the formation of so-called ‘black films’ between the lamellae of draining soap films). Neutron scattering studies have provided experimental evidence that such ‘black films’ can also occur between adhesive emulsion droplets (Poulin, Nallet, Cabane, & Bibette, 1996). Fig. 19 summarises the different steps of the process. As the droplets are forced together in the polymer solution (position 1) the liquid film between them is thinned (position 2) leaving less room for the polymer molecules, which require a finite volume of solvent to remain hydrated. At a certain point polymer molecules are forced out of the closing gap creating a very thin region between the droplets (position 3). At this point the droplet surfaces spontaneously jump closer together causing the jump-in events seen in the force curves. Once created this thin region quickly expands with time (position 4) pushing out polymer solute as it does so. This expansion of the very thin region can continue even whilst the drops are being pulled apart since, for a while at least, the region is still being subjected to a squeezing force by the deformed droplets. The work required to separate the droplets now becomes dominated by the area of this very thin film because the hydrodynamic suction created by this capillary-like film is very large. This explains the correlation between the jump-in point and the magnitude of the final pull-off adhesion seen in the force data: A slower approach speed allows the polymer to escape from the closing gap between the droplets earlier in the cycle, and subsequent expansion of this depleted region is given more time to proceed, resulting in a greater force being required to finally separate the droplets.

**Fig. 20:**
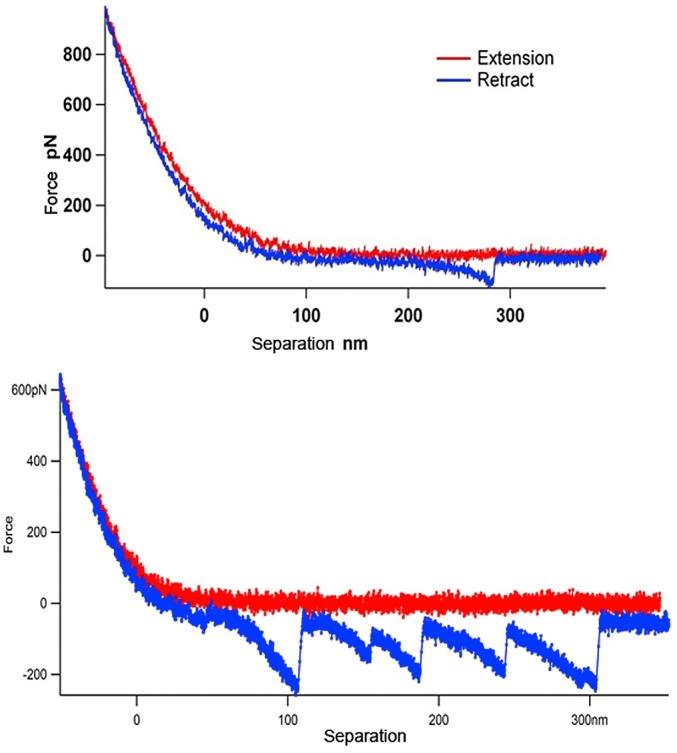
Association of pectin chains upon retraction of SBP coated droplets. (Top panel) in 4 mM CaCl2 (bottom panel) de-esterified SBP.

The final examination of the effect of SBP emulsified droplets was the potential for partial coalescence as the pectin chains can link together in certain conditions forming coagulated strings of the droplets which can have a significant effect on the physical properties of such emulsions. Normally the extension of the pectin chains into the bulk aqueous phase will lead to a steric repulsion between droplets as proposed by (Leroux et al., 2003). Manipulating the experimental bulk conditions promoted inter-chain association of the pectin (Fig. 20). In the presence of calcium ions, events characteristic of single molecule polymer stretching are observed in the retraction data at points beyond droplet separation (upper panel). Such bridging studies showed evidence for both single and multiple polymer stretching events following droplet separation (lower panel).

Finally, another significant set of AFM colloidal studies have been carried out at the University of Melbourne. In addition to the AFM force measurements they have created mathematical models of most of the aspects; deformability effects (Carnie et al., 2005), velocity effects (Webber et al., 2008), ionic strength effects (Clasohme et al., 2007), viscosity effects (Dagastine et al., 2010), polymer depletion effects (Browne et al., 2015b, Browne et al., 2015a) and characterisation of the profiles of thin liquid films between oil droplets and air bubbles (Vakarelski et al., 2010) and lamellae in foams.

## 4 Conclusions

The use AFM as a microscopic tool is now used widely to tackle problems in food and biological science. A key advantage is the ability to observe structure and structural heterogeneity at the molecular level, under ‘near natural’ conditions. Although widely used, the method is still not routine, and methodologies need to be modified or developed to suit particular biological samples. Use has led to new understanding of complex food systems with resultant applications. The use of the AFM as a force transducer is emerging as a new tool in food science, which can assist and expand the role as a microscope. For example whereas interactions between larger molecules such as proteins and polysaccharides can be visualised, force measurements are needed to identify and characterise binding of smaller molecules such as in oligosaccharide-protein interactions, which could not be seen directly by AFM. Similarly the ability to probe interactions between large objects, such as air bubbles or oil droplets offers the possibility to extend the imaging and understanding of interfaces into investigating their effect on the pair-pair interactions responsible for foam or emulsion stability. In addition, given an understanding of the changes in interfacial structure during complex processes such as digestion it ought to be possible to determine their effect on the stability and breakdown of food structures on consumption. Thus the combined use of the techniques offers routes to the design of novel food structures to tackle problems in health, dietary choice and nutrition.
